# Differential proteomics for studying action mechanisms of traditional Chinese medicines

**DOI:** 10.1186/s13020-018-0223-8

**Published:** 2019-01-08

**Authors:** Yi-Yao Yang, Feng-Qing Yang, Jian-Li Gao

**Affiliations:** 1 School of Chemistry and Chemical Engineering, Chongqing University, Chongqing, 401331 People’s Republic of China; 2 Academy of Chinese Medical Sciences, Zhejiang Chinese Medical University, Hangzhou, 310053 Zhejiang People’s Republic of China

**Keywords:** Differential proteomics, Traditional Chinese medicines, Action mechanisms, Identification

## Abstract

Differential proteomics, which has been widely used in studying of traditional Chinese medicines (TCMs) during the past 10 years, is a powerful tool to visualize differentially expressed proteins and analyzes their functions. In this paper, the applications of differential proteomics in exploring the action mechanisms of TCMs on various diseases including cancers, cardiovascular diseases, diabetes, liver diseases, kidney disorders and obesity, etc. were reviewed. Furthermore, differential proteomics in studying of TCMs identification, toxicity, processing and compatibility mechanisms were also included. This review will provide information for the further applications of differential proteomics in TCMs studies.

## Background

**Fig. 1:**
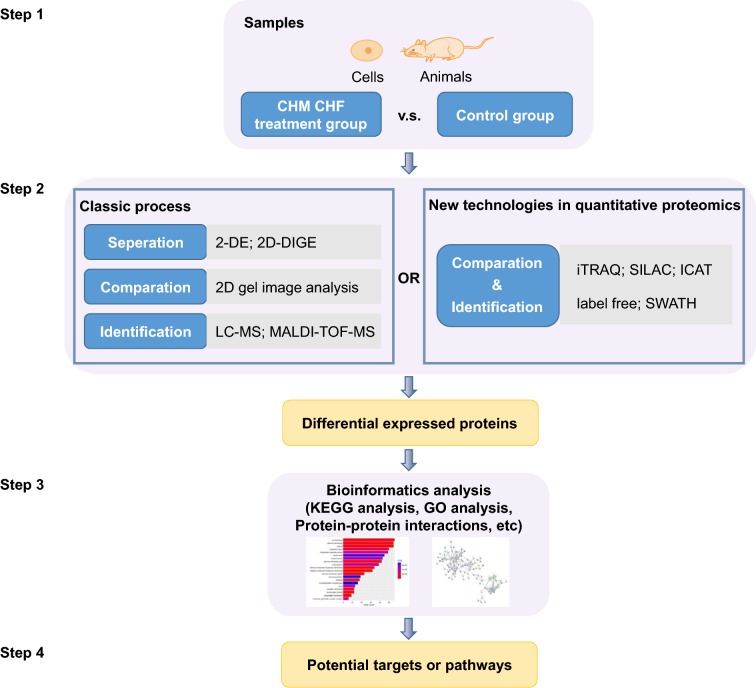
Schematic diagram of the experimental procedure for differential proteomics in studying of TCMs

Differential proteomics, which is also known as comparative proteomics or functional proteomics, studies the changes of proteome in different physiological or pathological states between two or more samples for the analysis of important life processes or major diseases in order to find out the key different proteins that are regarded as markers for qualitative and functional analysis [1, 2]. The classic process for differential proteomics in studying of traditional Chinese medicines (TCMs) is separation–comparison-identification (Fig. 1). To begin with, proteins are extracted from cells or animal models with/without TCM treatment. For separating these proteins, two-dimensional gel electrophoresis (2-DE) or two-dimension difference gel electrophoresis (2D-DIGE) are generally employed. After that the protein spots on the gel are compared and partly selected to be identified with mass spectrometry (MS). Alternatively, several new technologies in quantitative proteomics not only identify an enormous amount of proteins expressed in different states, but also accurately quantify their abundance. Isobaric tags for relative and absolute quantification (iTRAQ), which is the most widely used high-throughput technology integrating identification and quantification, makes the analysis of differential proteome easier and more efficient. In addition, labelling technologies such as stable isotope labeling with amino acids in cell culture (SILAC) and isotope coded affinity tag (ICAT), as well as label-free sequential window acquisition of all theoretical mass spectra (SWATH) are also used. Finally, differential expressed proteins can be found, following by bioinformatics analysis to find the connotation from their differences that can be indexed to potential targets or pathways.

Differential proteomics has been used to study TCMs for over a decade, and recently was developed rapidly. Most studies were mainly focused on the mechanisms of TCMs in treating diseases at the protein level, and looked for possible therapeutic targets of drug action. In the past, Liu and Guo [3] summarized the applications of proteomics in the mechanistic study of TCMs from 2004 (the first paper published) to 2011. In 2014, Lao et al. [4] summed up the mechanistic studies of TCMs in treating neurological disorders, cancers, cardiovascular diseases, diabetes and inflammation by using proteomics. And Ji et al. [5] reviewed the proteomic studies on the therapeutic mechanisms of TCMs (~ 2015) based on the perspectives of clinical researches, and in vitro or in vivo experimental animal models.

In this paper, the applications of differential proteomics in studying of TCMs, including the mechanistic studies of TCMs in treating diseases, TCMs identification, as well as the toxicity, processing and compatibility mechanisms studies of TCMs that can further broaden the understandings of TCMs, were summarized and discussed.

## Differential proteomics for exploring the action mechanisms of traditional Chinese medicines

**Fig. 2:**
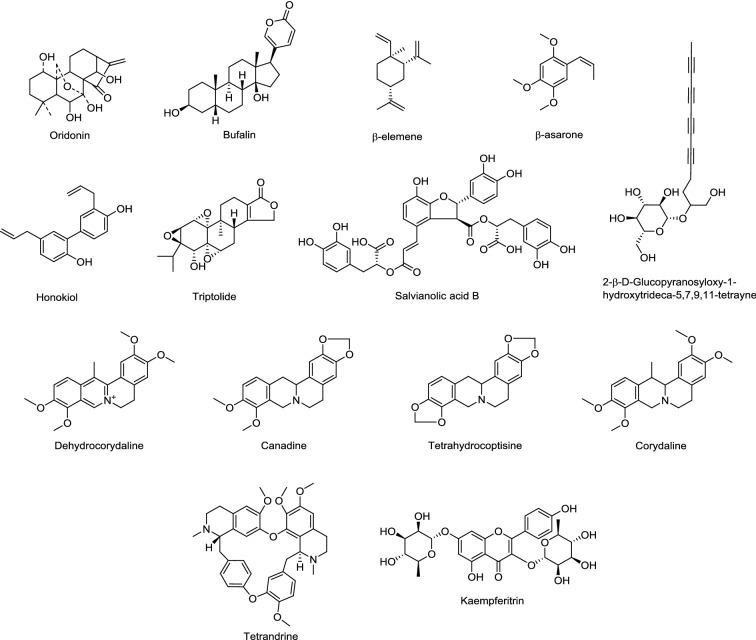
Chemical structures of main monomers involved in this paper

As a complex system of chemical components, TCMs involve multiple processes through regulating multiple targets. Studying on their action mechanisms has been a difficulty for researchers. Notably, the regulation of TCMs at protein level can be visualized by using proteomic technologies, through the analysis of the functions of significantly differential expressed proteins or further studying the pathways involved. Differential proteomics provides a practical and effective strategy for searching the action targets of TCMs, and improves understanding the therapeutic effects of TCMs at molecular level. As summarized in Table 1, differential proteomics approach had been applied in exploring the action mechanisms of TCMs for the treatment of cancers, cardiovascular diseases, diabetes, liver and kidney diseases, wound and obesity, etc. TCM monomers involved in these experiments are shown in Fig. 2.

**Table 1:** Differential proteomics in exploring the action mechanisms of TCMs

Diseases	Drugs	Potential key targets or signaling pathways	Proteomics methods	Ref.
Cancers				
Osteosarcomas	Bufalin	Heat shock 27 kDa protein	2-DE and MALDI-TOF/TOF–MS	[6]
T-cell acute lymphocytic leukemia	2-β-d-Glucopyranosyloxy-1-hydroxytrideca-5,7,9,11-tetrayne	Mitochondrial function (Parkinson disease protein 7, voltage-dependent anion-selective channel protein 2 and peroxiredoxin 3) and cell death (BH3 interacting domain death agonist and Lamin-B1 protein)	2-DE, MALDI-MS/MS and LC–ESI–MS/MS	[7]
Thyroid cancer	Honokiol	Glyceraldehyde-3-phosphate dehydrogenase, tubulin alpha-1A chain, alpha-enolase, 78 kDa glucose-regulated protein, proliferating cell nuclear antigen	2D-DIGE and MALDI-TOF–MS	[8]
Hepatocarcinoma	Oridonin	Heat shock 70 kDa protein 1, serine-threonine kinase receptor-associated protein, translationally-controlled tumor protein, stress-induced phosphoprotein 1, inorganic pyrophosphatase, trifunctional purine, chromobox protein homolog 1, glycyl-tRNA synthetase, poly(rC)-binding protein 1	2-DE and MALDI-TOF–MS/MS	[9]
Multiple myeloma	Oridonin	Stathmin, dihydrofolate reductase, pyruvate dehydrogenase E1β	2-DE and MALDI-TOF–MS/MS	[10]
Gastric cancer	β-Elemene	Bcl-2-associated transcription factor 1, Bcl-rambo, p21-activated protein kinase-interacting protein 1, S100 calcium binding protein A10, etc.	iTRAQ and LC–MS/MS	[11]
Glioblastoma	β-Asarone	Heterogeneous nuclear ribonucleoprotein H1 (H) isoform CRA b, heterogeneous nuclear ribonucleo-protein A2/B1 isoform CRA a, ubiquitin carboxyl-terminal hydrolase isozyme L1, cathepsin D	2-DE and MALDI-TOF/TOF–MS/MS	[12]
Lung cancer	Triptolide	Ribosome biogenesis in eukaryotes, spliceosome, mRNA surveillance pathway	iTRAQ and Nano LC–MS/MS	[13]
	β-Elemene	Peroxiredoxin-1	2D-DIGE and MALDI-TOF–MS	[14]
Cardiocerebrovascular diseases				
Cardiovascular disorders (antiplatelet)	Notoginsengnosides	Growth factor receptor-bound protein 2, thrombospondin 1, thioredoxin, Cu–Zn superoxide dismutase, Parkinson disease protein 7, peroxiredoxin 3, thioredoxin-like protein 2, ribonuclease inhibitor, myosin regulatory light chain 9, tubulin alpha 6, laminin receptor 1, potassium channel subfamily V member 2	2-DE and MALDI-TOF–MS/MS	[15]
	Salvianolic acids	LIM domain protein CLP-36, copine I, myosin regulatory light chain 9, coronin-1B, heat shock 70 kDa protein 2, heat shock 70 kDa protein 8, heat shock 70 kDa protein 9, peroxiredoxin 3, 3-mercaptopyruvate sulfurtransferase, fibrinogen gamma polypeptide, cytoplasmic dynein intermediate chain 2C, aminopeptidase P 1	2-DE and MALDI-TOF–MS/MS	[16]
	Salvianolic acid B	Integrin α2β1	2-DE and MALDI-TOF–MS/MS	[17]
	Olive oil	γ-Actin, Rho GDP-dissociation inhibitor 1, annexin A5, integrin αIIb, protein disulphide isomerase-related protein 5, fibrinogen gamma chain precursor, syntaxin-7, serine/threonine protein phosphatase	2-DE and LC–ESI–MS	[18]
	Rhizoma Corydalis	P2Y purinoceptor 12, Gαi signalling pathways	2-DE and MALDI-TOF–MS/MS	[19]
	Dehydrocorydaline, canadine	P2Y purinoceptor 1, 12 (dehydrocorydaline); G protein-coupled receptor protease-activated receptor 1 (canadine)	2-DE and MALDI-TOF–MS/MS	[20]
Cerebral IR injury	Tetrandrine	78 kDa glucose-regulated protein, Parkinson disease protein 7 and hypoxia up-regulated protein 1	2-D DIGE, MALDI-TOF/TOF–MS	[21]
	Bu-*Yang* Huan-Wu Decoction	Albumin, fibrinogen alpha chain, transferrin, calcium/calmodulin-dependent protein kinase type II alpha chain, glycogen synthase kinase 3, microtubule-associated protein tau, metabotropic glutamate receptor 5, guanine nucleotide-binding protein G (i), GDP dissociation inhibitor and 3-hydroxybutyrate dehydrogenase	iTRAQ and LC–MS/MS	[22]
	Tao-Hong Si-Wu Decoction	Superoxide dismutase [Cu–Zn], sulfiredoxin, glutathione *S*-transferase alpha-2, glutamate-cysteine ligase regulatory subunit, NAD(P)H dehydrogenase [quinone] 1, heme oxygenase 1	2-DE and MALDI-TOF/TOF–MS	[23]
Cardiac IR injury	Salvianolic acids, notoginsengnosides	Protein disulfide-isomerase, myosin light polypeptide 3, eukaryotic translation elongation factor 2 (SAs); apolipoprotein A-I, ubiquinol-cytochrome c reductase core protein, 60 kDa heat shock protein, prohibitin (NG)	2-DE and MALDI-TOF/TOF–MS	[24]
Liver diseases				
Liver injury	Yin-Chen-Hao-Tang	Zinc finger protein 407, haptoglobin, macroglobulin, alpha-1-antitrypsin, transthyretin, vitamin D-binding protein, prothrombin	2-DE and MALDI-TOF/TOF–MS	[25]
Liver fibrosis	Fu-Zheng Hua-Yu Recipe	Aldehyde dehydrogenase 1 family member A1, vimentin isoform CRA b, gamma-actin, vimentin, fructose-bisphosphate aldolase B, Aldo–keto reductase family 1 member D1, *S*-adenosylhomocysteine hydrolase isoform, Heat shock protein 90	2-DE and MALDI-TOF–MS	[26]
	Fu-Zheng Hua-Yu Recipe	Uridine diphosphate-glucuronosyltransferase 2A3, cytochrome P450 2B1 and cytochrome P450 3A18 in retinol metabolism pathway	iTRAQ and LC–MS/MS	[27]
	*Bupleurum marginatum* Wall.ex DC	Uridine diphosphate-glucuronosyltransferase, adenylate kinase isoenzyme 1, thioredoxin 1, acyl-CoA oxidase 2, glycogenin 1, alpha serine/threonine kinase, acyl-CoA synthetase medium-chain family member 1, carbonyl reductase family member 4	iTRAQ and LC–MS/MS	[28]
Wound healing				
Skin trauma	Radix Angelicae Sinensis	Triosephosphate isomerase, microtubule-associated protein RP/EB family member1, nucleoside diphosphate kinase B, glutathione *S*-transferase P, translationally-controlled tumor protein, peroxiredoxin, Parkinson disease protein 7, annexin A2	2-DE and LC–MS/MS	[29]
	Radix Lithospermi	Microtubule-associated protein RP/EB family member1, chloride intracellular channel protein 1, nucleoside diphosphate kinase A, phosphorylated signal protein P38, eukaryotic translation initiation factor 5A-1	2-DE and LC–MS/MS	[30]
	*Agrimonia pilosa, Nelumbo nucifera, Boswellia carteri,* pollen typhae (ANBP)	β-2-Glycoprotein 1, histidinerich glycoprotein, myosin family, keratin, extracellular matrix proteins	iTRAQ, HPLC and LC–MS/MS	[31]
	Radix Astragali, Radix Rehmanniae	Annexin A1, annexin A2, plasminogen activator inhibitor 1	2-DE and MALDI-TOF/TOF–MS	[32]
Diabetes				
T2DM	Yi-*Qi*-Yang-*Yin*-Hua-Tan-Qu-Yu Recipe	Cell division control protein 42 homolog, Ras homolog gene family member A	iTRAQ, 2D LC–MS/MS	[33]
	Xiaoke Pill	Angiotensinogen, alpha-1-antitrypsin, paraoxonase and fibulin	iTRAQ, MRM, 2D-LC, MALDI TOF/TOF–MS	[34]
	Kaempferitrin	Insulin-like growth factor-binding protein 2 and insulin-like growth factor-binding protein 4, low-density lipoprotein receptor, C-type mannose receptor 2, adipocyte enhancer-binding protein 1, Mannan-binding lectin serine protease 1	Dimethyl peptide labeling, LC–MS/MS	[35]
TCM deficiency syndrome				
Kidney-*yin* deficiency syndrome	Liu-Wei Di-Huang Granule	Retinol binding protein 4, transthyretin, apolipoprotein, Complement C4-B	2-DE and MALDI-TOF–MS	[36]
Kidney-*yang* deficiency syndrome	Jin-Kui Shen-*qi* Pill	Wnt signaling pathway, adherens junction, neurotrophin signaling pathway, B cell receptor signaling pathway, chemokine signaling pathway, PPAR signaling pathway, Fc gamma R-mediated phagocytosis, mitogen-activated protein kinase signaling pathway	iTRAQ-LC–MS/MS and UPLC-Q-TOF-HDMS	[37]
*Yin*-deficiency-heat syndrome	Zhi-Bai Di-Huang Granule	Zinc-alpha-2-glycoprotein, C-reactive protein, complement C1q subcomponent, mannose-binding protein C, l-selectin, plasminogen and kininogen-1	iTRAQ and 2D LC–MS/MS	[38]
Other diseases				
Hyperlipidemia	Yin-Chen Wu-Ling Powder	Apolipoprotein E, serum albumin, transthyretin and vitamin D-binding protein; T-kininogen, complement C3, C4, C4b-binding protein alpha chain, Igλ-2 chainC, mannose-binding protein C, hemopexin and fibrinogen-like protein 1	2-DE and MALDI-TOF–MS	[39]
Obesity	Taeumjowi-tang	Fatty acid synthetase, adenosine monophosphate-activated protein kinase/acetyl CoA carboxylase pathway	2-DE and MALDI-TOF–MS	[40]
Depression	Shen-Zhi-Ling tablet	Von Willebrand factor, protein Z-dependent protease inhibitor, alpha-2-macroglobulin, apolipoprotein C-III	label-free and LC–MS/MS	[41]
Chronic obstructive pulmonary disease	Bu-Fei Yi-Shen formula	Oxidative stress and focal adhesion pathway	iTRAQ and Nano LC–MS/MS	[42]
Fever	Bai-Hu-Tang	F-actin, coronin, nicotinamide adenine dinucleotide phosphate oxidase, major histocompatibility complex class I	iTRAQ and LC–MS	[43]
Aging	Red ginseng	Ubiquitin carboxyl-terminal hydrolase isozyme L1, heat shock 70 kDa protein, fructose-bisphosphate aldolase	2-DE and MALDI-TOF–MS	[44]

2-DE, two-dimensional electrophoresis; MALDI-TOF–MS, matrix-assisted laser desorption ionization time-of-flight mass spectrometry; iTRAQ, isobaric tags for relative and absolute quantification; LC–ESI–MS, liquid chromatography electrospray ionisation tandem mass spectrometry; 2D-DIGE, two-dimension difference gel electrophoresis; HPLC, high performance liquid chromatography; UPLC-Q-TOF-HDMS, ultra-performance liquid chromatography combined with quadrupole time of fight high definition mass spectrometry; IR, ischemic–reperfusion

### Cancer

As the exponentially rise in the global cancer burden, it already becomes an extremely urgent problem to control the development of cancers [45]. As TCMs have a long history in the treatment of various cancers, many studies have confirmed the therapeutic effects of Chinese herbal medicine (CHM) and Chinese herbal formula (CHF) on cancer in entire stages with the guidelines of TCM theories [46]. In recent years, many studies on differential proteomics analysis of monomers from anticancer TCMs have been carried out, towards hepatocarcinoma, bone tumor and gastric cancer, etc. Differential proteins provided clues that related mechanisms had connections with directly and/or indirectly affecting the multiple hallmark capabilities of cancer cells, such as tenacious vitality, unlimitedly proliferation, invasion and metastasis, etc.

Inducing apoptosis is an effective way to kill cancer cells thus against their vitality. Bufalin, the active ingredient of Chansu, was found to inhibit human osteosarcoma cell growth and induced G2/M arrest and apoptosis. Twenty-four differentially expressed proteins after bufalin treatment were identified by a comparative proteomics approach. And the heat shock 27 kDa protein, which plays a vital role in oncotherapy for its anti-apoptotic and tumorigenic properties, was most dramatically down-regulated [6]. Therefore, inhibition of heat shock 27 kDa protein expression played a key role in bufalin-induced apoptosis in osteosarcoma cells. In another study, effects of 2-β-d-glucopyranosyloxy-1-hydroxytrideca-5,7,9,11-tetrayne (GHTT), isolated from *Bidens pilosa,* on proteins expression in Jurkat T cells was investigated by 2-DE coupled with MS analysis. Results indicated that GHTT treatment can upregulate thirteen proteins involved in signal transduction, detoxification, metabolism, energy pathways and channel transport, as well as downregulate nine proteins, including thioredoxinlike proteins, BH3 interacting domain death agonist (BID protein involving apoptosis), methylcrotonoyl-CoA carboxylase beta chain and NADH-ubiquinone oxidoreductase. Furthermore, two pathways in Jurkat cells including mitochondrial dysfunction and apoptosis were predicted by bioinformatics analysis based on the data obtained from the differential proteomics approach [7]. Suppressing proliferation of cancer cells is another way for inhibitory effect of active compound. Honokiol from *Magnolia officinalis* was found to inhibit tumor cell growth, and its possible mechanism on thyroid cancer cell line was investigated by differential proteomics analysis [8]. Results indicated that honokiol altered the expression of 178 proteins, most of which showed as down-regulation and involved in cellular metabolic process, such as dysregulation of cytoskeleton, protein folding, transcription control and glycolysis. Combined with network analysis, glyceraldehyde-3-phosphate dehydrogenase, tubulin alpha-1A chain, alpha-enolase, 78 kDa glucose-regulated protein and proliferating cell nuclear antigen might be the potential targets in thyroid cancer therapy. In reality, some TCM monomers were found to play both proliferation-inhibiting and death-promoting roles in different pathways in tumor cells. *Rabdosia rubescens* is a representative anticancer eat-clearing and detoxicating herb, and its main bioactive compound oridonin was found to be able to fight various types of cancers [47]. The action mechanism in treating hepatocarcinoma of oridonin was investigated by proteomic tools [9]. Proliferative inhibition effect of oridonin was related with inhibiting telomerase and tyrosine kinase (chromobox protein homolog 1 and glycyl-tRNA synthetase), and arresting cells in G2/M phase (serine-threonine kinase receptor-associated protein, translationally-controlled tumor protein, stress-induced phosphoprotein 1, inorganic pyrophosphatase, poly(rC)-binding protein 1). While serine-threonine kinase receptor-associated protein, heat shock 70 kDa protein 1, trifunctional purine may responsible for cell apoptosis. Furthermore, oridonin was also found to modulate the expression of seven proteins in human multiple myeloma cell line [10]. Especially, there were three target proteins were found for the potential treatment of multiple myeloma. Dihydrofolate reductase was positively involved in folate metabolism, which indirectly inhibited DNA replication and induced tumor cell apoptosis. And stathmin was overexpressed in malignancy contributed to tumor angiogenesis and progression, pyruvate dehydrogenase E1β might reverse the Warburg effect.

TCM monomers can also inhibit tumor cell invasion and metastasis. Based on the differential proteomics study, underlying anticancer mechanisms of β-elemene that extracted from *Curcuma wenyujin* on gastric cancer cells were pro-apoptosis and metastasis-resistant effects [11]. The remarkably overexpressed protein p21-activated protein kinase-interacting protein 1 inhibited tumorigenesis and metastasis by targeting cancer-related protein P21-activated protein kinase 1, while significantly under-expressed protein S100 calcium binding protein A10 contributed to the weakening of tumor invasion and metastasis by influencing on the intracellular calcium signal. Moreover, two altered proteins (Bcl-2-associated transcription factor 1 and Bcl-2-like protein 13) both have pro-apoptosis activities.

In reality, the discovered mechanisms are greatly complex, since TCM-regulated proteins are involved in a variety of cellular process. β-asarone, as likely as the active compound contributes to the effect of Rhizoma Acori Graminei on central nervous system disorders, may has the possibility as therapeutic strategies on glioblastoma with quite high degree of malignity. To compare the proteomic difference associated with anti-tumor effects of β-asarone, human glioblastoma cell was used as model [12]. Four evidently altered proteins, heterogeneous nuclear ribonucleoprotein H1 (H), isoform CRA b, heterogeneous nuclear ribonucleo-protein A2/B1, isoform CRA a, ubiquitin carboxyl-terminal hydrolase isozyme L1 and cathepsin D were considered to be key protein targets, which fell into diverse molecular functions and could lead to cytotoxicity. On the other hand, there were evidences about how triptolide (from *Tripterygium wilfordii*) exerts its broad-spectrum antitumor activity on lung adenocarcinoma cells by engaging to iTRAQ [13]. Results indicated that 312 dysregulation proteins participated in action mechanisms of triptolide. The down-regulated proteins were involved in most significant pathways including ribosome biogenesis in eukaryotes, spliceosome and mRNA surveillance pathway, which all take part in the core process of gene expression and protein synthesis. While most of up-regulated proteins supported energy needs for the apoptosis process.

It is worth mentioned that TCM also can play a supporting role during radiotherapy of cancer. For example, β-elemene decreased reactive oxygen species (ROS) clearance in A549 cells through inhibiting the expression levels of radiation-induced peroxiredoxin-1, suggesting that it could enhance the radio-sensitivity of lung cancer cells [14].

### Cardiocerebrovascular diseases

**Fig. 3:**
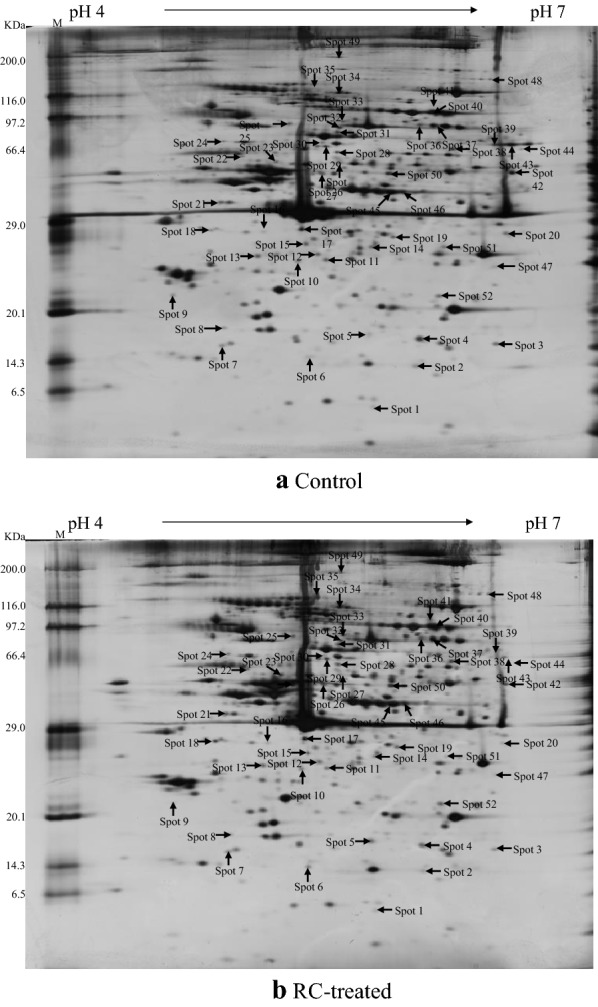
The 2-DE proteome images of control (**a**) and RC-treated (**b**) platelets. The differentially expressed protein spots were shown by the arrows Reproduced from ref [19] with permission from the authors

Antiplatelet and anticoagulant therapies play a crucial role in the prevention and treatment of cardiocerebral vascular diseases, which are tightly associated with blood stasis syndromes. And a variety of TCMs for promoting blood circulation and removing blood stasis have significant anti-platelet aggregation effects [48]. Therefore, differential proteins based on platelet proteomics were usually investigated to explore the action mechanisms for this kind of TCMs. For example, notoginsengnosides (NG) (derived from *Panax notoginseng*), changed 12 proteins expression in rat washed platelet, which indicated that its anti-platelet aggregative activity was attributed to scavenging ROS and modulating platelet activation, as well as reorganizing cytoskeleton structure [15]. Salvianolic acids (SAs) showed similar mechanism with NG, and SAs-modulated proteins also implicated in platelet adhesion, signal transduction and other functions [16]. In reality, there existed significant relationship between integrin and platelet function. As an important protein target of salvianolic acid B (SB), integrin α2β1 could bind with SB directly and SB triggered signal cascades was changed [17]. While after treated by olive oil extract, integrin aIIb/b3 could regulate platelet structure and aggregation, coagulation and apoptosis, and signaling [18]. In our previous study, ethanol extract of Rhizoma Corydalis (RC) has been investigated for its anti-platelet aggregation mechanism by differential proteomic analysis [19]. And 52 altered proteins (Fig. 3) were involved in platelet activation, oxidation stress and cytoskeleton structure. The potential direct target protein P2Y purinoceptor 1, as a crucial player, participated in signaling cascades network of RC during platelet aggregation. And the binding between RC extract and P2Y purinoceptor 1, followed with mediating Gαi signaling pathways, may contribute to the anti-platelet effect of RC. Furthermore, Tan et al. [20] had carried out further studies to elucidate the mechanisms underlying actions of dehydrocorydaline and canadine, which are the main anti-platelet aggregation active ingredients in RC. The key direct target proteins of dehydrocorydaline were two ADP receptors: P2Y purinoceptor 1 and P2Y purinoceptor 12. Dehydrocorydaline might exerted its impact mainly by acting on cytoskeleton-related proteins and RhoA/Myosin light chain 2 signaling pathway. For canadine, it may interacts with the G protein-coupled receptor protease-activated receptor 1, and modulate the phosphatidylinositol 3-kinases signaling pathway.

In common ischemic diseases, cerebral and cardiac ischemic–reperfusion (IR) injury are resulted from blood circulation disorder. Some of TCM monomers, CHM and CHF, such as tetrandrine, *Salvia miltiorrhiza*, *Panax notoginseng*, Bu-*Yang* Huan-Wu Decoration (BHD), Tao-Hong Si-Wu Decoction (THSWD) have been shown to have protective effects on ischemic diseases. Since series of biological activities of tetrandrine represents the potential application future in stroke therapy, Lin et al. [21] established middle cerebral artery occlusion mice model, from which thirty tetrandrine-modulated proteins were identified by using 2D-DIGE and MALDI-TOF-MS. Three key proteins including 78 kDa glucose-regulated protein, Parkinson disease protein 7 and hypoxia up-regulated protein 1 might be linked to neuroprotection effect, wherein 78 kDa glucose-regulated protein and Parkinson disease protein 7 treat stroke by preventing cell damage during ischemic brain injury, but the relationship between hypoxia up-regulated protein 1 and tetrandrine was not clear. The TCM *Salvia miltiorrhiza* and *Panax notoginseng* were usually employed for the treatment of ischemic cardiovascular diseases. To investigate their molecular mechanisms, Yue et al. [24] tentatively examined the effects of SAs, NG and their combination in rat models of IR injury, and 15 IR-related differentially regulated proteins were found. These results showed that SAs and NG had distinct regulatory effects on proteins involved in lipid metabolism, muscle contraction, heat shock stress, while their combination showed better effects for regulating both targets of SAs and NG. Chen et al. [22] studied a CHF used in treating *qi* deficiency and blood stasis syndrome caused by stroke, BHD. By analyzing brain tissue proteome from cerebral IR-induced stroke mouse model, it was depicted that BHD can decrease the expression of albumin, fibrinogen alpha chain, transferrin to reduce the blood–brain barrier breakdown, and the effects of modulated calcium/calmodulin-dependent protein kinase type II alpha chain, glycogen synthase kinase 3 and microtubule-associated protein tau embodied in neuroprotection, and suppressed excitotoxicity were ascribed to metabotropic glutamate receptor 5, nucleotide-binding protein G (i) and GDP dissociation inhibitor. In addition, uniquely BHD-regulated protein 3-hydroxybutyrate dehydrogenase indicated an involvement of enhancing energy metabolism. Compared to BHD, THSWD was also used for treating cerebrovascular diseases with different molecular mechanism. Qi et al. [23] found that THSWD could change the proteome of rat pheochromocytoma cells, hence it mediated protective effect on cerebral IR injury. They speculated that the protection effect of THSWD might regulated partly by six of Nrf2-driven phase II enzymes, which were validated in transcription level by real-time PCR.

### Liver diseases

Yin-Chen-Hao-Tang (YCHT) has often been used to treat liver diseases clinically. Using 2-DE and MALDI-TOF/TOF–MS analysis, Sun et al. [25] investigated the effects of YCHT on liver proteins in bile duct ligated rats and found that the expressions of fifteen proteins were modulated by YCHT, including zinc finger protein 407, haptoglobin, macroglobulin, alpha-1-antitrypsin, transthyretin, vitamin D-binding protein, and prothrombin. These proteins might be the most possible direct targets of YCHT, which involved in metabolism, energy generation, chaperone, etc. On the other hand, various liver injuries can lead to hepatic fibrosis during the process of sustained wound healing [49]. Chinese herbal formula Fu-Zheng Hua-Yu Recipe (FZHY) has been shown the effect of anti-hepatic fibrosis. To investigate its action mechanisms, Xie et al. [26] employed 2-DE and MALDI-TOF–MS on the analysis of proteome of normal, dimethylnitrosamine- induced fibrogenesis and FZHY-treated rats. Eight differential proteins in normal and FZHY-treated rats both showed reverse trends with model group, among which vimentin and gamma-actin had a link with inhibiting activation of hepatic stellate cell or epithelial-to-mesenchymal transition in liver cells, and the other six proteins were associated with stress response and metabolisms of retinoic acid, carbohydrate and bile acid. In a recent study, Dong et al. [27] discovered 255 genes and 499 proteins that all differently expressed by using microarray and iTRAQ. The three potential key proteins (uridine diphosphate-glucuronosyltransferase 2A3, cytochrome P450 2B1 and cytochrome P450 3A18) and three important pathways (retinol metabolism, metabolism of xenobiotics by cytochrome P450, and drug metabolism) were found via bioinformatics methods, which further elucidated the therapeutic mechanisms and pharmacological effects of FZHY. Effects of another anti-liver fibrosis TCM *Bupleurum marginatum* Wall.ex DC (BM) on protein expression in liver fibrosis rat was also investigated by iTRAQ [28]. The identified proteins were classified and involved in embracing drug metabolism, oxidative stress, biomolecular synthesis and metabolism, etc. Besides, based on compound-target network analysis, eight key targets (uridine diphosphate-glucuronosyltransferase 2A3, adenylate kinase isoenzyme 1, thioredoxin 1, acyl-CoA oxidase 2, glycogenin 1, alpha serine/threonine kinase, acyl-CoA synthetase medium-chain family member 1, carbonyl reductase family member 4) were excavated, as well as key active compounds (triterpenoid saponins and lignans) were identified.

### Wound healing

Chinese herbal medicine for wound healing has a long history and a relatively comprehensive theoretical system in China. Rising attention has been paid to the mechanisms of wound healing on molecular level. Shiunko, which is an effective CHF for external application to promote granulation and get rid of putrid necrosis, composes of two major components Radix Angelicae Sinensis (RAS) and Radix Lithospermi (RL) in promoting the wound healing process. Respectively, their mechanisms of action were studied by Hsiao et al. [29] through proteomics analysis. By using 2-DE, the proteins expression of human embryonic skin fibroblast treated with RAS were examined, and fifty-one remarkably up/down-regulated proteins were found, of which the functions were ascribed to promotion of glycolysis, enhancement of cell mobility and increase of antiapoptosis, etc. Functions of these proteins revealed that action mechanisms of RAS might be related to increasing the viability of cells during the wound healing process. Subsequently, as to RL, there were some similar effects brought by same or different regulated proteins contribute to molecular basis compared to RAS, but there had differences to a certain extent [30]. They embodied in cell mobility (down-regulation of chloride intracellular channel protein 1) and cell viability (up-regulation of nucleoside diphosphate kinase A, eukaryotic translation initiation factor 5A-1 and phosphorylated signal protein P38). Additionally, Chen et al. [31] found that herbal mixture ANBP (*Agrimonia pilosa*, *Nelumbo nucifera*, *Boswellia carteri*, and Pollen Typhae) aided the wound recovery at different healing stages by observing changes of skin proteome in trauma model rats. At length, ANBP-modulated proteins took part in immune and defense response, vascular system restoration, hemostasis and coagulation regulation and other processes at the early stages, while the formation of muscle tissue, hair, epidermis and extracellular matrix were promoted at the later stages. A modified formula (named NF3) composed of Radix Astragali and Radix Rehmanniae, exerted significant effects of wound healing and proangiogenesis separately in vivo and in vitro. Tam et al. [32] found that the treatment with NF3 modulated the expression of cytoskeleton regulatory proteins at the proteome level, such as annexin A1, annexin A2 and plasminogen activator inhibitor 1 in relation to proangiogenesis.

### Diabetes

TCMs also have potential clinical applications for the treatment of type 2 diabetes mellitus (T2DM). Yi-*Qi*-Yang-*Yin*-Hua-Tan-Qu-Yu Recipe (YQYYHTQY), which composes of eight CHMs, is an antidiabetic CHF. Study indicated that four of YQYYHTQY-regulated serum proteins had connections with diabetes, blood and behavior based on STRING analysis, of which two significantly decreased proteins (cell division control protein 42 homolog and Ras homolog gene family member A) belonged to small GTPase, were the crucial nodes involved in positive regulation of cytokinesis and response to glucose. Therefore, these two proteins might be the targets of YQYYHTQY on T2DM therapy [33]. However, diabetes treatments are often accompanied with adverse reactions, such as hypoglycemia. As Xiaoke Pill is beneficial in treating diabetic hypoglycemia, Zhang et al. [34] employed a modified iTRAQ strategy to study its mechanism. According to the variation patterns of protein abundance, the way of Xiaoke Pill affecting serum proteome was of difference to common anti-diabetic drug glyburide. And angiotensinogen, alpha-1-antitrypsin, paraoxonase and fibulin were presumed to be linked with its anti-diabetic effect. In addition, kaempferitrin extracted from the leaves of *Cinnamomum osmophloeum* and *Bauhinia forficata* also has potential antidiabetic effects. In distinct secretomes of kaempferitrin-treated astrocytic cell line, 32 regulated proteins were associated with insulin-related signaling, inflammation process, cholesterol metabolism. Among them, insulin-like growth factor-binding protein 2, insulin-like growth factor-binding protein 4 and low-density lipoprotein receptor were most likely to be antidiabetic-related proteins. And C-type mannose receptor 2, adipocyte enhancer-binding protein 1 and mannan-binding lectin serine protease 1 might inhibit the inflammatory response by keeping pro-inflammatory cytokines as normal [35].

### TCM deficiency syndrome

Studies have also been carried out to find the underlying mechanism of TCM on deficiency syndrome. By evaluating Liu-Wei Di-Huang Granule treatment in vitro fertilization pre-embryo transfer in infertility women with kidney-*yin* deficiency syndrome, Lian et al. [36] explored four possible underlying targets involved were retinol binding protein 4, transthyretin, apolipoprotein, as well as complement C4-B. The Jin-Kui Shen-*Qi* Pill (JSP), also called Ba-Wei Di-Huang Granule, exerts remarkable therapeutic efficacy in protecting against kidney-*yang* deficiency syndrome (KYDS) clinically. Zhang et al. [37] demonstrated proteomics and metabolomics methods to detect the differentially expressed serum proteins between JSP-treated and controlled rat models. It was therefore revealed that JSP had influence on KYDS by the regulation of metabolism-related proteins involved in wnt signaling pathway, adherens junction, as well as neurotrophin signaling pathway, etc. And about the differential proteomic studies of *yin*-deficiency-heat (YDH) syndrome treatments using CHF Zhi-Bai Di-Huang Granule (ZDG), which is equivalent to Liu-Wei Di-Huang Granule combined with Cortex Phellodendri and Rhizoma Anemarrhenae. Liu et al. [38] investigated the molecular mechanism of ZDG’s efficacy in nourishing *yin* and decreasing internal heat. ZDG-regulated proteins were found to be involved in antigen processing and presentation (zinc-alpha-2-glycoprotein), complement activation (C-reactive protein, complement C1q subcomponent, and mannose-binding protein C) and regulating the inflammatory response (L-selectin, plasminogen, and kininogen-1). Therefore, regulating the immune response to strengthen immunity might be the way of ZDG ameliorating YDH syndrome.

Obesity is a chronic metabolic disease caused by a variety of factors. People with obesity have fat metabolic disorder, which can lead to hyperlipidemia. The ways for researchers observing therapeutic effects of TCMs on obesity or hyperlipidemia are usually through measuring adipose tissue weight [50], serum parameters (such as leptin, cholesterol and triglyceride content) [51], etc. And differential proteomic provides a reference at the protein level. Li et al. [39] utilized comparative proteomic approach for the molecular mechanism research of Yin-Chen Wu-Ling Powder on hyperlipidemic model rats. Serum proteome was analyzed and twelve significantly altered plasma proteins were identified. The finding suggested that efficacy of positively modulating lipid levels had affinity with the functions of differently expressed proteins, that includes regulating lipid metabolism, improving coagulation functional disturbance, regulating immune and inflammatory responses, and mediating substance transport. Another anti-obesity herbal medicine Taeumjowi-tang (TH) consisting of eight herbs has traditionally been used in Korea. Kim et al. [40] identified the proteins differentially expressed in hepar of TH-treated obesity model rats utilizing proteomic and western blot analysis, and deduced that TH improved lipid metabolism through modulating fatty acid metabolizing proteins involved in obesity and hepatic injury, with the involvement of adenosine monophosphate-activated protein kinase, acetyl CoA carboxylase and fatty acid synthetase.

The proteomics was also employed for uncovering the molecular mechanisms of TCM treatments on other diseases. For example, von Willebrand factor, protein Z-dependent protease inhibitor, alpha-2-macroglobulin, and apolipoprotein C-III were considered as potential targets for Shen-Zhi-Ling in treating depression [41]; Bu-Fei Yi-Shen formula might alter the expression of proteins involved in oxidative stress and focal adhesion to treat chronic obstructive pulmonary disease [42]; Bai-Hu-Tang might fight against lipopolysaccharide fever syndrome by up-regulating F-actin, coronin, nicotinamide adenine dinucleotide phosphate oxidase and major histocompatibility complex class I [43]; Red ginseng could modulate antioxidant-related proteins ubiquitin carboxyl-terminal hydrolase isozyme L1, heat shock 70 kDa protein, fructose-bisphosphate aldolase against aging [44], etc.

## Identifications of traditional Chinese medicines by differential proteomics approach

Nowadays, there were many methods used to characterize and identify of TCMs, such as UPLC-QTOF/MS combined with chemometrics to find out unique markers for Radix Polygoni Multiflori from different geographical areas [52], quality control of *Lycium chinense* and *Lycium barbarum* cortex by HPLC using kukoamines as markers [53]. Although small molecules were usually been used as quality control markers for TCMs, plant origin proteins, which have various kinds of bioactivities [54], also facilitate the identification of TCM. Differential proteomics can be used to find characteristic proteins in Chinese herbal samples that differ in origins, species, medicinal parts, as well as wild types and artificial cultivation types, thus it provides information of material basis and plays the role of identification.

To this day, there have been a number of studies on the different proteins of fungi TCMs for identification and quality control, due to its biological activities and abundance. A representative and valuable fungal Chinese herb is Cordyceps (*Ophiocordyceps sinensis*). In the study of *O. sinensis*, Zhang et al. [55] used 2-DE and MALDI-TOF/TOF–MS to compare proteins of *O. sinensis* samples that five were collected from different habitats (three from China, two respectively from Nepal and Bhutan) and other four were different fungal specimens with similar shape; They found that distribution of *O. sinensis* protein spots among the five regions has no striking differences, and two specific proteins OCS_04585 and b-lactamase domain-containing protein were identified, while the comparison results between four fungal specimens showed that there was only one common protein (protein-eliciting plant response-like protein) existed. A more extensive research about habitats was carried out by Li [56] to find differentially expressed protein of *O. sinensis*. The abundance and number of proteins varied greatly among 26 habitats from Sichuan, Tibet and Qinghai provinces. To find out the correlation between natural *O. sinensis* protein and its origin, by using cluster analysis towards protein spots, the samples were divided into two categories: those from Tibet and from Qinghai. This study provided a meaningful reference for finding protein markers of *O. sinensis* from different habitats. On the basis of previous studies on protein markers, Tong et al. [57] conducted deeper research towards *O. sinensis* samples collected from four production regions and other four counterfeit samples. The differences in protein of *O. sinensis* from Yunnan, Sichuan, Tibet and Qinghai provinces were reflected in the distribution and concentration, and the proteome of authentic *O. sinensis* and its counterfeits existed great differences. Total 22 characteristic proteins were identified, of which IP4 can be used as a putative target in the indirect ELISA developed by them. In addition, Zhang et al. [58] found 165 proteins differed significantly between the samples of natural and artificial cultivation. As the supply of natural *O. sinensis* cannot meet the market demand, it is of importance to investigate quality formation of artificially-cultivated *O. sinensis* and supply valuable references and guidance for its artificial cultivation. About other fungi TCMs, Li et al. [56, 59] analyzed proteins in *Ganoderma lucidum* and *Morchella vulgaris* by gel electrophoresis, wherein fourteen samples of *G. lucidum* from different habitats or seven samples of *M. volgaris* from three habitats with different processing methods all showed that the number and abundance of proteins were distinct.

There were also some proteomic researches on other herbal medicines. The difference proteins among four medicinal aloe (*Aloe barbadensis* Miller, *A. vera* L. var* chinensis* (Haw.) Berger, *A. ferox* Miller and *A. arborescens* Miller) were investigated in Fan’s study [60]. There was a certain amount (about 51% to 62%) of differential proteins between the four medicinal aloes. Among them, the ran-binding protein 1 homolog c-like, actin, NAD-dependent malate dehydrogenase and cinnamyl alcohol dehydrogenase existed in *A. barbadensis*; the alpha tubulin subunit, isoflavone reductase-like proteins presented in *A. vera* var *chinensis*; and the auxin-induced protein PCNT115-like isoform 1 was found in *A. arborescens*. In another study, by using proteomic methods, proteins from Oriental ginseng and American ginseng, different parts of Oriental ginseng, cultured cells of Oriental ginseng were compared to find out marker proteins [61]. Nine common protein spots existed in all parts of two species, while the protein spots AM1 and KM1 were found only in main roots of Oriental ginseng and American ginseng, respectively. Cultured cells contained much more alkaline proteins than Oriental ginseng. In other herbal medicines, Hua et al. [62] established a omic-based strategy to comprehensively reveal and accurately measure gene and protein expression in naturally- and artificially-cultivated *Pseudostellaria heterophylla*. And 71 of 332 proteins were remarkably altered. The differences could be the cause that artificially-cultivated *P. heterophylla* was more capable in the ability to respond to stress and the catabolism of oxidoreductasesm, but weak in carbohydrate metabolism of hydrolases, carbohydrate and cellular amino acid metabolisms of transferases.

Moreover, as one of the important resources of TCMs, animal medicines are particularly rich in proteins and peptides which enables differential proteomics to become a very potential tool for their quality identification. Sodium dodecyl sulphate–polyacrylamide gel electrophoresis and 2-DE were conducted to distinguish three gelatinous Chinese medicines: Asini Corii Colla (ACC), Testudinis Carapacis ET Plastri Colla (TCPC), Cervi Cornus Colla [63]. The range of protein molecular weight was as varied as Colla species, but the spots were dispersed in the gel which brought about difficulty in protein identification. Therefore, these protein spots were treated with trypsinase. With the identification of characteristic polypeptide fragments using MALDI-TOF/TOF–MS and Nano-LC Orbitrap MS, nineteen characteristic proteins were found in ACC while seven in TCPC. Furthermore, Xue et al. [64] developed shotgun proteomics and bioinformatics strategy that can identify differential collagen in ACC made from the skin of donkey, horse, pig or cattle. Six specific peptides from the collagen of four kinds of ACC as skin markers were found, such as ^497^GPTGEPGKPGDK^508^ for donkey, ^422^GASGPAGVR^430^ and ^497^GPSGEPGKPGDK^508^ for horse, ^422^GPTGPAGVR^430^ for pig, ^781^GEAGPSGPAGPTGAR^795^ and ^352^GEGGPQGPR^360^ for cattle. The strategy can be applied to detect the adulteration of non-donkey species sensitively.

## Miscellaneous

Studies on TCM toxicity are beneficial to establish a scientific assessment system to guarantee the safety in clinical TCM medication. Differential proteomics can be used to dig the toxicity mechanisms of TCMs by comparing TCM-treated and control groups to find abnormally regulated proteins. Xu et al. [65] observed changes in abundances of embryo proteins in model rats treated with *Pinellia ternata* (Thunb.) Breit. They used proteomic analysis and identified 153 differential expressed proteins that enriched in pathways of oxidative phosphorylation metabolism and neurodegenerative diseases. Among them, 37 specific proteins mainly inhibited the process of nervous system development, including brain development and neuron development, which associated with fetal nervous system abnormalities. Li et al. [66] tested the liver toxicity of saikosaponins isolated from Radix Bupleuri in mice and established a relationship among dose, time course and hepatotoxicity. In addition, 487 proteins, which involved in pathways of lipid metabolism, protein metabolism, macro molecular transportation, cytoskeleton structure and response to stress, showed distinct differential expression patterns before and after saikosaponins treatment and might induce liver injury.

Processing is a characteristic pharmaceutical technology in TCMs, which has positive effects such as increasing effect, reducing toxicity and alleviating drug property, etc. But the principle of processing is still unclear, and there are lacks of effective quality control standards during processing [67]. Differential proteomics provides a new idea for it, and starts from two aspects: changes in the proteins of TCMs before and after processing; changes in the molecular mechanism after its actions on cells or animals. To study the mechanism of reducing toxic effects on intestines between Semen Euphorbiae and its processed product-Semen Euphorbiae Pulveratum (SEP) in KM mice, Zhang et al. [68] performed iTRAQ and LC–MS/MS analysis and uncovered two differential expressed proteins as key inflammatory biomarkers, of which angiopoietin-4, signal transducer and activator of transcription 1 attenuate inflammatory response via affecting Janus kinase 2/signal transducer and activator of transcription 3 signaling pathway, and angiopoietin/angiopoietin-1 receptor signaling pathway respectively, after treated with SEP. Traditional fried process on *Pilose antler* has the function of removing blood residue and antisepsis, but it is likely to cause loss of active ingredients. Jin [69] found that 37 of the differential *Pilose antler* proteins involved in anti-fatigue and metabolism were destroyed, and author recommended that freeze drying process with protective agent was a better choice. Xu [70] discovered that proteins of the processed *Bombyx batryticatus* were obviously less than that of crude drug, which indicated stir-baking with bran may degrade the protein. And thirteen different proteins were identified. Fu et al. [71] carried out comparative proteomic analysis on *Eisenia fetida* processed by sun- and freeze-drying. Five fibrinolytic proteases that possibly related to thrombolytic activity were identified, and their total abundance of freeze-dried earthworms was dramatically higher than that of sun-dried.

Compatibility is another feature of the theoretical system of TCM, which embodies the concept of wholism and differentiation criteria. The interaction between compatible medicines includes mutual reinforcement and opposite, mutual restraint and detoxication, mutual assistance and inhibition according to ‘Shen Nong’s Herbal Classic’. Recently, the study on the compatibility by using differential proteomics has received certain attention. The proteomic study on Qi-Shen-Yi-*Qi* formula (QSYQ) has been well explained its compatibility mechanism [72]. QSYQ constituted by *Panax notoginseng*, *Salvia miltiorrhiza*, *Astragalus membranaceus* and *Dalbergia odorifera*, which are individually classified as monarch herb, minister herb, assistant herb and guide herb. The CHF exerts treating effects for ‘*Qi*-deficiency, blood stasis’ coronary heart disease. Studies were carried out on rats divided into the control, each medical herb alone, combined treatment groups, and myocardial infarction model group. The number of differentially regulated proteins of the four drugs was 17, 16, 15 and 15, respectively. These results indicated that effects of each drug had different emphasis in angiogenesis and reduced energy consumption, anti-oxidation and anti-adhesion, promotion of angiogenesis, promotion of microangiogenesis. Miao et al. [73] explored the effects of single herb Radix Scutellariae, Rhizoma Coptidis and their herbal pair in the liver tissue of rats. Total 78 proteins expressed differently were associated with drug metabolism, energy metabolism, signal transduction and cytoskeleton. These toxicity-related proteins showed a certain degree of difference among three groups, which provided a useful reference for future research. Differential proteomic analysis provides a fresh look at compatibility study of herbal pair. As to studies on TCM incompatibility, Yu [74] discovered the possible mechanism of the effects of glycyrrhizic acid and genkwanin on reducing or increasing toxicity, both of which are the active representative compounds of the incompatible herbal pair, Radix Glycyrrhiza and Flos Genkw, respectively. Two treatment groups had forty-six overlaps up-regulated proteins and seventy-nine down-regulated proteins, and these proteins regulated the pathways related to glycerophospholipid metabolism, virus infection, pathogenic bacteria infection and cell tight junctions.

## Conclusion

Protein is the specific practitioner of life activities, the dynamic change shows the characteristic life activity in real time, which close to life phenomena and essence [3]. The differential proteome focuses on the different proteins with a certain implication under the changes caused by different states, and extracts distinction from the whole, and produces the aggressive propulsion effects in the exploration of various mechanisms behind the TCM theory. In recent years, several reports have applied differential proteomics in TCMs researches. Among them, studies on therapeutic mechanism of TCMs take the majority, understanding the role of TCMs in the treatment of cancer, cardiovascular diseases, diabetes and so on has been growing. Not only that, differential proteomics has other applications in the TCMs identification, mechanism study of toxicity, processing and compatibility theory.

Although proteomic techniques have been rapidly developed, the promotion of technologies have been limited by high cost. For its high separation efficiency, 2-DE remains the mainstream technique for protein separation. However, 2-DE has characteristics of low sensitivity, time-consuming and complex operation, unable to be directly combined with MS, likewise incomplete identification of protein species, etc. Furthermore, even with advanced quantitative proteomics techniques, researchers still face challenges. iTRAQ as an example, the difficulty lies in complex preparations processes of samples (A, protein extraction; B, preliminary quantitative analysis; C, enzyme digestion; D, labelling; E, balanced mix), dealing with a great deal of MS information of labelled digested peptide. In addition, as a good partner of differential proteomics, bioinformatics methods can mine useful information from the mass of data (protein location, function, enriched pathway, and interaction network) for predicting the signaling pathways.

Up to now, as the existing researches were preliminary and partial, and the information obtained through proteomics techniques was still limited, which could suggest potential mechanisms but in-depth theoretical study was not enough. Conjunction with other omics technologies to collect multi-level information of molecules (e.g. genes, metabolites, etc.) has become an inevitable trend. Besides those top-down approaches, bottom-up approaches help TCM studies become more accurate and concentrated [75]. For example, hypotheses can be proposed on the basis of data analysis through network pharmacology [76], and then it could be used for complementing, testing and verifying mutually with the results of differential proteomic to find out TCM mechanisms effectively.

## Abbreviations

2D-DIGE: two-dimension difference gel electrophoresis
2-DE: two-dimensional gel electrophoresis
ACC: Asini Corii Colla
ANBP: *Agrimonia pilosa*, *Nelumbo nucifera*, *Boswellia carteri*, and Pollen Typhae
BHD: Bu-*Yang* Huan-Wu Decoration
BM: *Bupleurum marginatum* Wall.ex DC
CHF: Chinese herbal formula
CHM: Chinese herbal medicines
FZHY: Fu-Zheng Hua-Yu Recipe
GHTT: 2-β-d-glucopyranosyloxy-1-hydroxytrideca-5,7,9,11-tetrayne
ICAT: isotope coded affinity tag
IR: ischemic–reperfusion
iTRAQ: isobaric tags for relative and absolute quantification
JSP: Jin-Kui Shen-*Qi* Pill
KYDS: kidney-*yang* deficiency syndrome
LC–MS/MS: liquid chromatography tandem mass spectrometry
MALDI-TOF–MS: matrix-assisted laser desorption ionization time-of-flight mass spectrometry
MS: mass spectrometry
NF3: a modified formula composed of Radix Astragali and Radix Rehmanniae
NG: notoginsengnosides
QSYQ: Qi-Shen-Yi-*Qi* formula
RAS: Radix Angelicae Sinensis
RC: Rhizoma Corydalis
RL: Radix Lithospermi
ROS: reactive oxygen species
SAs: salvianolic acids
SB: salvianolic acid B
SEP: Semen Euphorbiae Pulveratum
SILAC: stable isotope labeling with amino acids in cell culture
SWATH: sequential window acquisition of all theoretical mass spectra
T2DM: type 2 diabetes mellitus
TCMs: traditional Chinese medicines
TCPC: Testudinis Carapacis ET Plastri Colla
TH: Taeumjowi-tang
THSWD: Tao-Hong Si-Wu Decoction
YCHT: Yin-Chen-Hao-Tang
YDH: *Yin*-deficiency-heat
YQYYHTQY: Yi-*Qi*-Yang-*Yin*-Hua-Tan-Qu-Yu Recipe
ZDG: Zhi-Bai Di-Huang Granule
